# Dialysis Access-Associated Steal Syndrome in a Hemodialysis Patient: A Case Report

**DOI:** 10.7759/cureus.105101

**Published:** 2026-03-12

**Authors:** Renato Abu Hana, Ruben G Ortiz Cordero, Oswaldo A Guevara Tirado, Grit A Adler, Vinicius Adami Vayego Fornazari

**Affiliations:** 1 Department of Radiology, University of Florida College of Medicine – Jacksonville, Jacksonville, USA

**Keywords:** arteriovenous fistula (avf), dialysis access complications, dialysis access steal syndrome, fistulography, hemodialysis vascular access

## Abstract

Dialysis access-associated steal syndrome (DASS) is an uncommon but potentially limb-threatening complication of hemodialysis arteriovenous access. DASS results from preferential shunting of arterial blood into the low-resistance access circuit, causing distal hypoperfusion and symptoms ranging from hand coolness and paresthesias to skin ulceration and tissue loss. We discuss the case of a 32-year-old patient with end-stage renal disease on hemodialysis who presented with prolonged bleeding after dialysis from their right upper-extremity arteriovenous fistula. The patient then underwent fistulography with endovascular treatment of venous outflow pathology and pseudoaneurysms. One month later, the patient developed worsening dialysis-associated forearm/hand pain and paresthesias. Repeat angiography demonstrated poor anterograde distal forearm perfusion with flow reversal into the fistula, absence of proximal arterial inflow stenosis, and restoration of distal perfusion with venous outflow compression, supporting the diagnosis of DASS. This case highlights the importance of recognizing dialysis-associated ischemic symptoms while reviewing a mechanism-based diagnostic approach used to guide management.

## Introduction

Arteriovenous fistulas (AVFs) are the preferred vascular access for long-term hemodialysis due to their durability and lower risk of infection [1-3]. Despite these advantages, arteriovenous (AV) accesses are still associated with clinically significant complications including stenosis, thrombosis, venous hypertension, aneurysmal degeneration, high-output cardiac physiology, and distal upper extremity ischemia [3,4]. Dialysis access-associated steal syndrome (DASS) is an uncommon, yet potentially limb-threatening complication that requires prompt recognition and targeted intervention to preserve both the extremity and access [5].

DASS typically occurs when arterial flow is diverted into the low-resistance access circuit, resulting in reduced hand perfusion [5-7]. In practical terms, the AV access creates an unintended low-resistance pathway that diverts blood flow to the fistula rather than the higher-resistance hand circulation. Recognized risk factors include older age, female sex, diabetes mellitus, peripheral artery disease, prior ipsilateral access procedures, and high-flow accesses [5,8]. Symptomatic DASS occurs in approximately 1-8% of AV accesses overall [5,7]. Among cases severe enough to require intervention, upper-arm (brachial-artery inflow) accesses are affected more frequently than forearm accesses (~4-9% vs. ~0.3-2%) [5]. Symptoms can vary from coldness and paresthesia during dialysis to severe hand ischemia causing constant pain at rest, neurologic deficits (weakness and numbness), skin ulcers, and tissue loss [5,7,8].

Management of DASS is typically driven by the severity of the patient’s symptoms and underlying etiology [5]. Furthermore, the treatment goal is primarily to restore distal perfusion and preserve a functional dialysis access when feasible [2]. Treatment options include correction of proximal inflow or distal outflow arterial disease via endovascular therapy, access flow reduction when a high-flow physiology is present, surgical revascularization, or access ligation or embolization when ischemia persists and salvage is not possible [2,7,8]. In this report, we present the case of DASS in the setting of recent endovascular intervention and discuss a systematic diagnostic approach and mechanism-based management strategy to improve patient outcomes. We also highlight key angiographic findings supporting steal physiology, including distal hypoperfusion with flow reversal into the fistula and improvement with venous outflow compression.

## Case presentation

Initial admission

A 32-year-old patient with a past medical history of end-stage renal disease (ESRD) on hemodialysis, liver cirrhosis due to chronic hepatitis C, lupus, congestive heart failure, chronic obstructive pulmonary disease, epilepsy, and uncontrolled hypertension presented to the hospital due to prolonged bleeding in their right arm AVF after their last dialysis session. The patient was reported to have required approximately 30 minutes of direct pressure to achieve hemostasis and had to stop the dialysis session about 15 minutes early. Vital signs upon admission demonstrated hypertension (160/124 mmHg) and tachycardia (108 beats per minute). Physical examination was unremarkable, and there was no evidence of active bleeding or signs of infection at the AVF site. Laboratory results were remarkable for anemia, mild thrombocytopenia, leukopenia, elevated creatinine, elevated blood urea nitrogen, a decreased estimated glomerular filtration rate (eGFR), and hyperkalemia (Table 1).

**Table 1:** Lab values on first and second admissions.

Test	Results (1st admission)	Results (2nd admission)	Reference Range	Unit
Hemoglobin	8.3	11.3	14.0–18.0 (male), 12.0–16.0 (female)	g/dL
Platelet count	120	92	140–440	×10³/µL
White blood cell count	2.87	4.51	4.5–11	×10³/µL
Creatinine	9.77	6.25	0.67–1.17	mg/dL
Urea nitrogen	56.9	34	6.0–22.0	mg/dL
Estimated glomerular filtration rate	5	9	≥60	mL/min/1.73m^2^
International normalized ratio	1.0	1.0	0.8–1.1	Ratio
Sodium	138	139	135–145	mmol/L
Potassium	6.5	5.4	3.4–4.5	mmol/L

**Figure 1:**
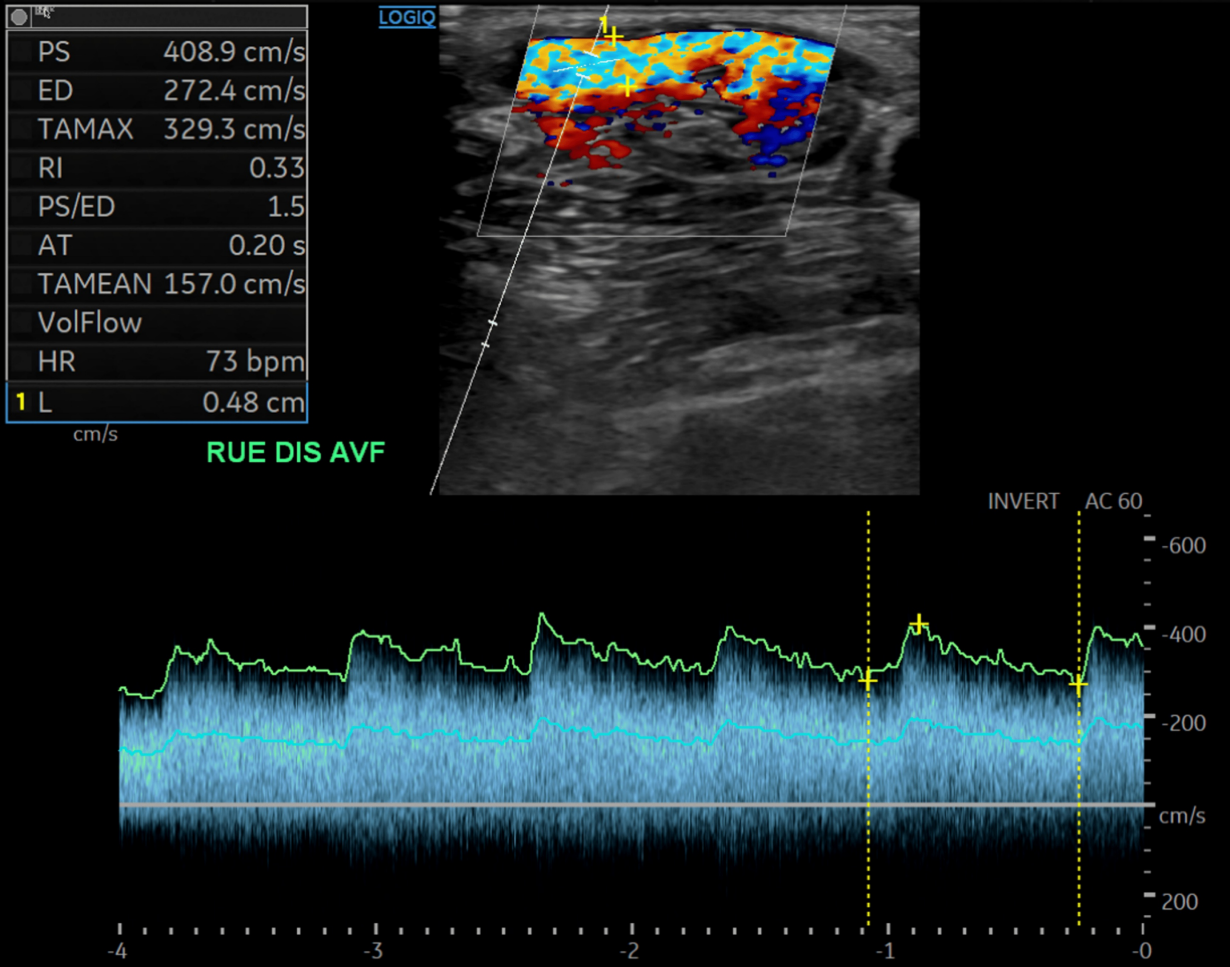
Doppler ultrasound of a right upper extremity arteriovenous fistula. Color Doppler demonstrates turbulent flow within the arterialized outflow vein. Spectral Doppler reveals elevated velocities (PSV 409 cm/s) and low resistive index (RI 0.33) with continuous diastolic flow, compatible with high-flow fistula physiology and suspicious for focal outflow stenosis. PSV, peak systolic velocity

**Figure 2:**
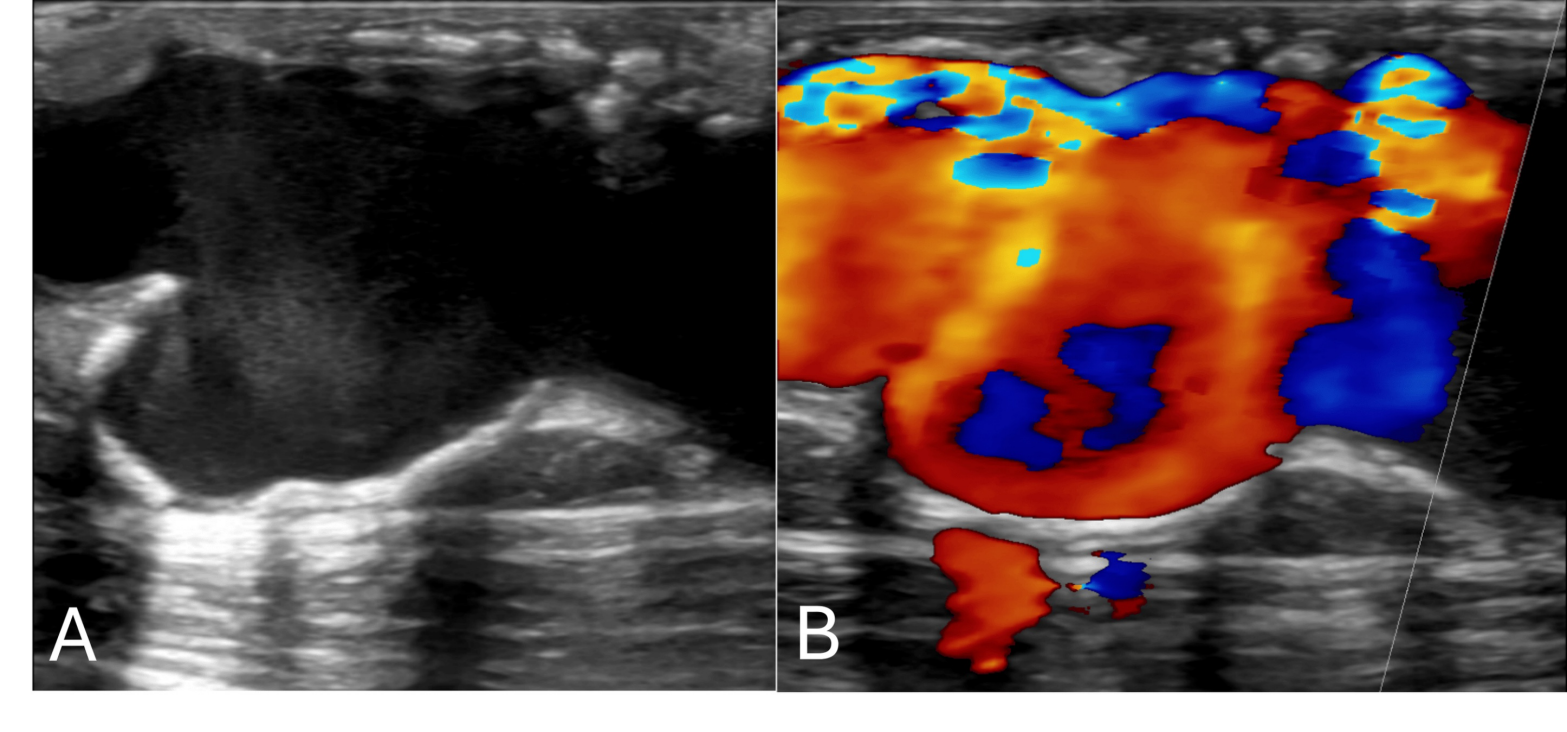
Ultrasound evaluation of the venous outflow of an arteriovenous fistula. (A) A grayscale ultrasound image demonstrates an irregular, aneurysmal dilation of the venous outflow segment. (B) A color Doppler image of the same region shows turbulent, disorganized flow within the aneurysmal segment, consistent with high-flow fistula physiology and possible associated hemodynamic disturbance.

A targeted Doppler ultrasound of the right upper extremity AVF was performed, revealing a patent access with normal spectral waveforms. However, there was evidence of narrowing at the fistula venous outflow segment with elevated velocities, suggestive of stenosis and large irregular aneurysmal dilation (Figures 1, 2). Given their prolonged bleeding after dialysis, as well as ultrasound findings, interventional radiology was consulted for a fistulogram with possible intervention.

**Figure 3:**
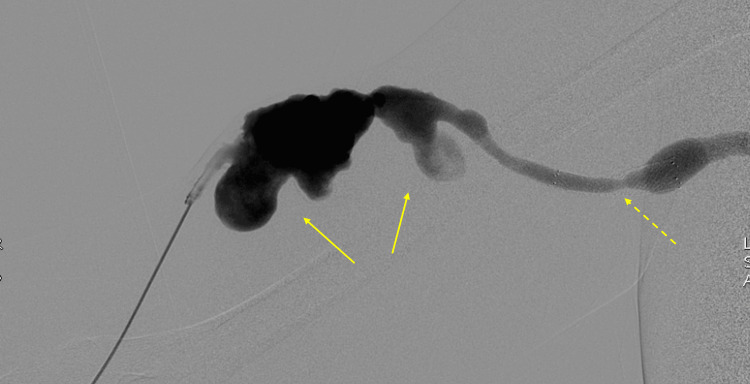
Digital subtraction angiography fistulogram of the arteriovenous fistula demonstrating irregular aneurysmal dilatation of the venous outflow segment (solid arrows) with associated moderate stenosis within the stent (in-stent stenosis) (dashed arrow).

**Figure 4:**
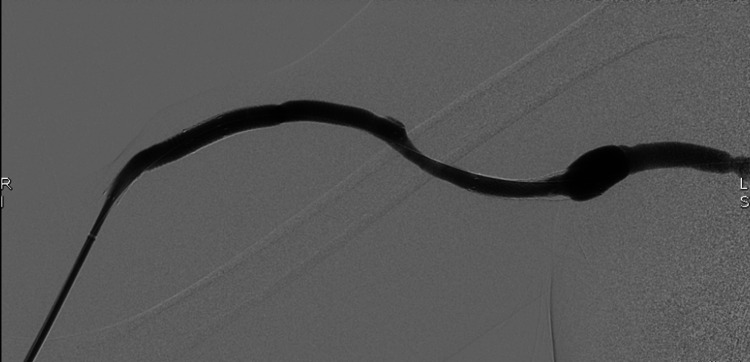
Post-treatment DSA fistulogram. Following covered stent placement, the DSA image demonstrates satisfactory exclusion of the aneurysmal dilation with no significant residual in-stent stenosis. DSA, digital subtraction angiography

**Figure 5:**
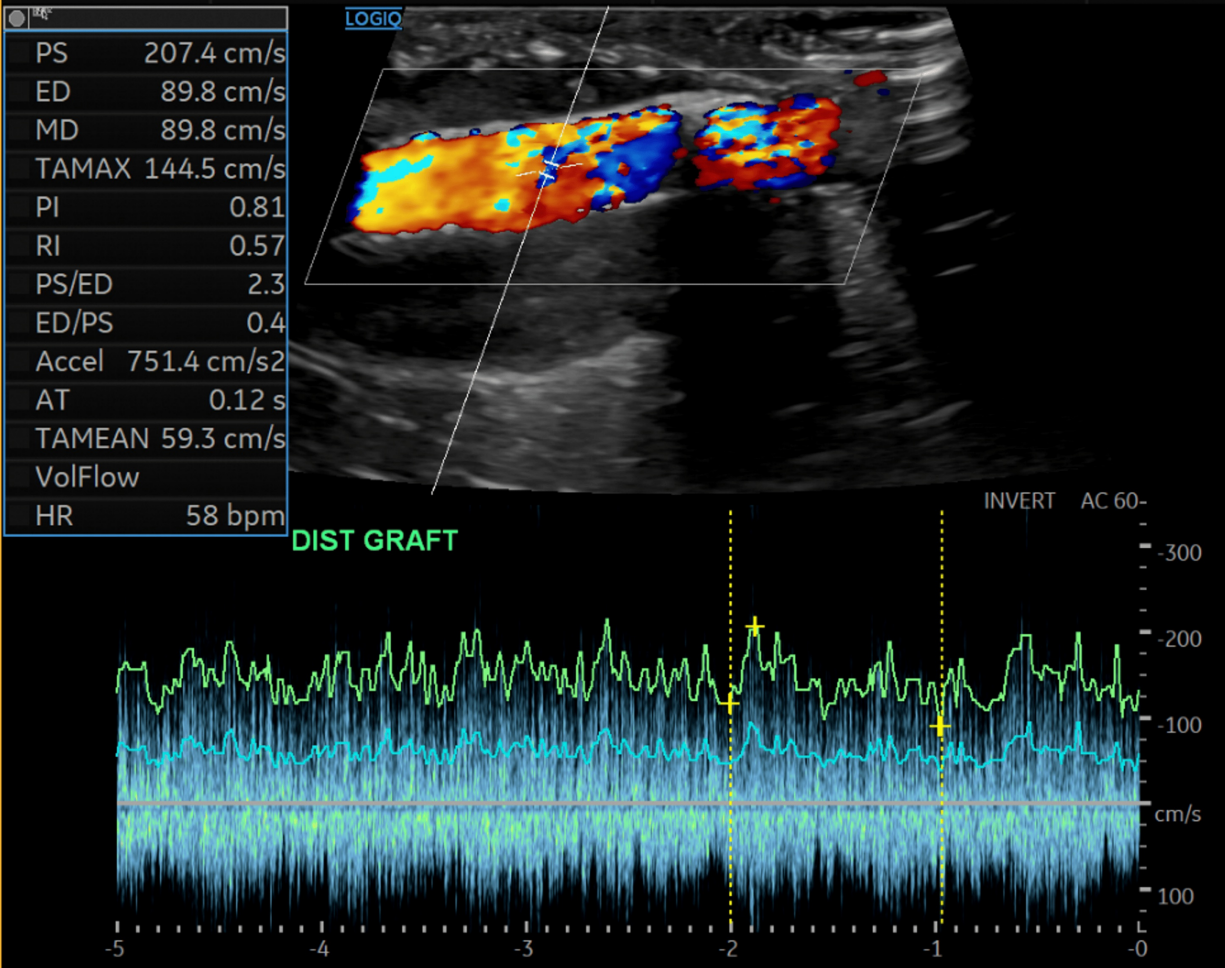
Post-intervention Doppler ultrasound of the arteriovenous fistula. Color Doppler demonstrates improved laminar flow within the distal fistula following stent placement. Spectral Doppler shows normalization of velocities compared with the pre-intervention study (PSV 207 cm/s, RI 0.57), consistent with improved hemodynamics. PSV, peak systolic velocity; RI, resistive index

After obtaining appropriate informed consent, the patient underwent right upper extremity AVF angiography. Standard sterile preparation was performed, and local anesthesia with 4 mL of 2% lidocaine was administered at the access site. Prophylactic cefazolin (Ancef) 2 grams intravenously (IV) was given prior to intervention, and systemic anticoagulation was achieved with heparin 3,000 units IV during the case. AVF angiography showed multiple pseudoaneurysms in the venous outflow associated with intrastent stenosis at the axillary vein (Figure 3). Intra-stent angioplasty was performed using a 7 mm × 40 mm Mustang OTW PTA balloon dilatation catheter (Boston Scientific, Marlborough, MA, USA). Following this, two Viabahn covered stents measuring 8 mm × 10 cm and 8 mm × 5 cm (W. L. Gore & Associates, Newark, DE, USA) were deployed in an overlapping fashion across the pseudoaneurysms within the venous outflow. Final angiogram demonstrated complete exclusion of the aneurysms and no residual stenosis in the vein outflow (Figure 4). A post-intervention Doppler ultrasound demonstrated improved hemodynamics with normalization of spectral parameters compared with the pre-intervention study (Figure 5). Overall, the patient tolerated the procedure well without any apparent signs or symptoms of distress. Post-intervention hemodialysis demonstrated an increase in achieved blood flow rate (BFR) from 500 mL/min pre-intervention to 800 mL/min post-intervention (BFR goal: 400 mL/min). The patient was monitored while in the hospital and later discharged on post-procedural day 3.

Second admission

Approximately one month later, the patient returned to the emergency department due to persistent (8/10 in intensity) pain around their AVF access site with associated paresthesia that worsened during their dialysis sessions. On evaluation, the access site appeared to be intact with a palpable thrill and present distal pulses. No signs of infection were visualized. Vital signs were remarkable for hypertension (144/105 mmHg). Laboratory results were remarkable for anemia, thrombocytopenia, elevated creatinine, elevated blood urea nitrogen, decreased eGFR, and hyperkalemia (Table 1). Targeted Doppler ultrasound of the right upper extremity access demonstrated an interim increase in peak systolic velocity at the distal anastomosis, concerning for stenosis with redemonstrated narrowing in this region. Additionally, there was revisualization of multiple occluded and partially thrombosed pseudoaneurysms along the midportion of the fistula. Interventional radiology was consulted for a fistulogram as there was concern for DASS. Appropriate informed consent was obtained for a right upper extremity fistulogram to assess access patency and distal perfusion.

Standard sterile preparation was performed. Local anesthesia with 2% lidocaine (10 mL subcutaneously) was administered at the access site. Moderate sedation and analgesia were provided during the procedure with midazolam 1.5 mg IV and fentanyl 85 mcg IV. Retrograde access was obtained with a micropuncture set. Using a 0.035″ Terumo Glidewire® and a 4-French Terumo Glidecath® (Terumo Corporation, Tokyo, Japan), the axillary artery was catheterized. Angiogram was performed, which showed patency of the arterial system, AV anastomosis, and the entire AVF with no significant venous outflow or central stenosis.

**Figure 6:**
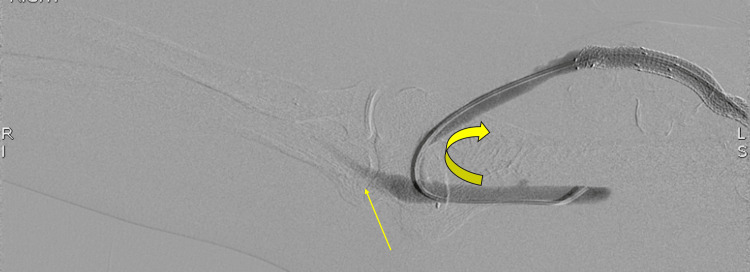
One-month follow-up digital subtraction angiography fistulogram. Selective contrast injection from the brachial artery demonstrates brisk preferential flow into the arteriovenous fistula (curved arrow) with minimal opacification of the native forearm arteries (straight arrow), demonstrating steal physiology.

**Figure 7:**
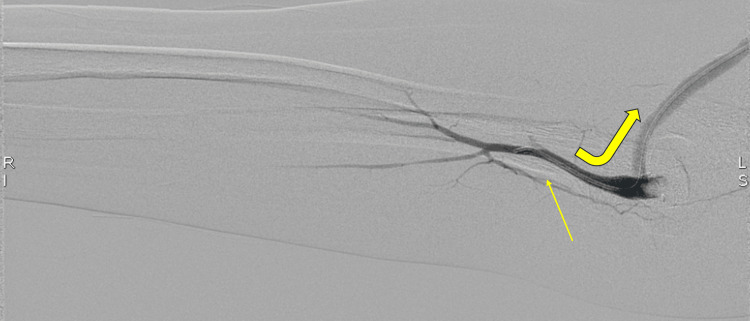
Digital subtraction angiography from the distal brachial artery. Injection distal to the arteriovenous anastomosis demonstrates retrograde flow with preferential opacification of the arteriovenous fistula (curved arrow) rather than the native forearm arteries (straight arrow), findings supportive of dialysis access-associated steal syndrome.

However, the angiogram revealed poor anterograde flow to the distal forearm (Figure 6). Following that, the catheter was placed at the distal brachial artery just beyond the AV anastomosis, and the contrast injection showed reversal flow to the AVF (Figure 7).

Finally, when compressing the venous outflow, the angiogram demonstrated restoration of the arterial inflow and distal arterial perfusion to the forearm and hand with immediate improvement of the patient’s symptoms.

The right upper extremity fistulogram was completed as planned without any immediate complications.

Given the patient’s clinical presentation and imaging findings, DASS was suspected, and a formal vascular surgery consultation was recommended for further evaluation and management. During evaluation by the vascular surgeon, the patient reported that the right upper extremity AVF had been created proximally approximately 1.5-2 years earlier. Since its creation, the fistula has required multiple salvage procedures, including angioplasty and thrombectomy for recurrent stenosis and thrombosis.

On further history obtained by vascular surgery, the patient reported that approximately three to six months after access creation, they began experiencing numbness, tingling, and pain in the right forearm and hand, which has persisted. These symptoms were described as intermittent but progressively worsened, particularly following the most recent intervention involving stent placement and angioplasty, which exacerbated symptoms, especially during dialysis sessions. At this time, the patient denied rest pain at baseline, tissue loss, nonhealing wounds, or skin discoloration.

Physical examination revealed numbness and tingling in the right upper extremity that worsened with repetitive or increased activity. Grip strength was preserved at 5/5, sensation remained intact, and there were no signs of proximal inflow disease or current limb-threatening ischemia.

Overall, the patient remained clinically stable and expressed a strong preference for outpatient management of the access-related issue. Access-preserving treatment options, including AVF revision and flow-reduction procedures, were discussed. The patient was scheduled for follow-up in the vascular surgery clinic, and hemodialysis was to continue via the current access site unless symptoms worsened. A concise summary of the clinical course is provided in Table 2.

**Table 2:** Timeline of clinical course, diagnostic findings, and management AV, arteriovenous; AVF, arteriovenous fistula; DASS, dialysis access-associated steal syndrome; HD, hemodialysis; US, ultrasound

Timepoint	Clinical Event	Key Findings	Management/Outcome
~1.5–2 years before presentation	Right upper-arm AVF created (outside facility)	-	-
~3–6 months after AVF creation	Onset of symptoms	Intermittent numbness/tingling and forearm/hand pain, worse during dialysis	Continued HD via AVF; symptoms persisted
Initial admission	Prolonged post-dialysis bleeding from AVF	US: venous outflow narrowing with elevated velocities; aneurysmal dilation	AVF angiography + venous outflow intervention (angioplasty + covered stents); discharged post-procedure day 3
~1 month later (second admission)	Worsening dialysis-associated forearm/hand pain + paresthesias	Angiography: poor antegrade distal forearm perfusion, flow reversal into AVF; perfusion improved with venous outflow compression	DASS suspected; vascular surgery consulted
Evaluation after second admission	Disposition/plan	No rest pain or tissue loss; preserved strength and pulses	Outpatient vascular surgery follow-up for access-preserving revision/flow-reduction planning; continue HD via current access unless symptoms worsen

## Discussion

DASS occurs in approximately 1-8% of patients with AV access, while clinically significant DASS requiring intervention only occurs in 0.3-2% of patients with forearm access and 4-9% in patients with upper arm access [5]. A systematic review found that ischemic steal events occur more predominantly in elderly patients with upper arm fistula [4]. Despite our patient’s young age, their history of multiple prior salvage procedures and upper-arm AV access site placed them at higher risk for access-related hand ischemia. Furthermore, laboratory abnormalities, including anemia and thrombocytopenia, were consistent with the patient’s chronic ESRD and liver disease and were not felt to be contributory to their ischemic symptoms (Table 1).

The pathophysiology of DASS is typically multifactorial and may involve proximal inflow stenosis, distal arterial disease affecting the radial, ulnar, or palmar circulation, excessive access flow (so-called “true steal”), and impaired collateralization or limited arterial adaptive capacity in patients with atherosclerosis or medial calcification [7]. In our patient, symptoms were primarily forearm/hand pain and paresthesias that were predominantly associated with their dialysis sessions, consistent with a non-limb threatening presentation of DASS.

Endovascular interventions, such as balloon angioplasty or stent placement, may precipitate or worsen steal symptoms by increasing flow through the access. By relieving venous outflow stenosis and lowering vessel resistance during an endovascular examination, access flow may increase, leading to new or exacerbated distal hypoperfusion in susceptible patients [7]. Therefore, assessment for DASS following such procedures is often recommended [7]. In our case, after multiple prior interventions, including stent placements and angioplasties, the patient experienced worsening dialysis-associated forearm/hand symptoms. Subsequent diagnostic angiography (Figures 6, 7) confirmed steal physiology in the absence of proximal arterial inflow stenosis, with restoration of distal perfusion during venous outflow compression. These findings, in conjunction with the patient’s intermittent, dialysis-associated symptoms, supported a hemodynamic steal physiology.

DASS can be graded from 0 to 3 depending on clinical presentation [1]. A grade 0 indicates that no steal is present, whereas grade 1 (mild) is characterized by peripheral coolness with minimal symptoms and augmented flow with occlusion of the access [1]. Grade 2 (moderate) reflects intermittent ischemia, typically occurring during dialysis or with exertion, likely seen in the presented case. Lastly, grade 3 (severe) is defined by ischemic rest pain or tissue loss [1]. Diagnosis of DASS starts with physical examination where manual compression of the access can lead to symptom relief or improvement in distal pulses [5,7]. Physiologic testing includes assessing the digit-brachial index (DBI) and digital systolic pressure, which, if abnormal, are strongly related to clinically relevant DASS [7,8]. A DBI of less than 0.6 and/or a digital pressure of less than 50-60 mmHg can be supportive thresholds for the diagnosis of DASS [7,8]. In a study by Goff et al., the presence of a DBI less than 0.60 was reported to be sensitive for identifying patients at risk of developing steal syndrome; however, it had poor specificity and positive predictive value [9]. Finally, the use of duplex ultrasound and angiography can help define treatable lesions and guide management [2]. Imaging the entire arterial tree with angiography, from the aortic arch to the palmar arch with and without access compression, is often recommended to avoid overlooking proximal inflow or distal runoff disease contributing to DASS [2]. While DBI and digital pressures can help quantify severity and guide management, these measurements were not obtained in our patient. However, objective post-intervention changes, including improved Doppler spectral parameters (peak systolic velocity/resistive index) and an increased achieved dialysis BFR, supported improved access hemodynamics. Diagnosis was further supported by angiography demonstrating clear distal hypoperfusion with flow reversal into the fistula and restoration of distal perfusion (with immediate symptom improvement) during venous outflow compression.

The management of DASS depends on the patient’s symptoms and mechanism of the disease [5]. Patients with mild symptoms such as intermittent hand coolness or numbness (occurring mostly during dialysis) without rest pain or tissue loss can be observed and managed conservatively with warm compresses and, in selected cases, vasodilators [5]. However, individuals who have more persistent or severe symptoms, including disabling pain during dialysis or exertion, rest pain, neurologic deficits, ulceration, or tissue loss, typically require invasive intervention [2,5]. Given our patient’s preserved strength, intact pulses, and absence of tissue loss or rest pain, immediate intervention was not indicated, and the patient’s preferences for an outpatient evaluation for access-preserving options were deemed appropriate.

Beyond symptom severity, selecting the appropriate intervention requires identifying the predominant hemodynamic mechanism of disease [5,7]. When arterial inflow or distal runoff disease contributes, treatment focuses on correcting proximal or distal arterial lesions with angioplasty and stenting or surgical bypass [5,8]. When high access flow is the primary cause, strategies aimed at reducing access flow such as calibrated banding (including MILLER), revision using distal inflow (RUDI), or other flow-limiting surgical revisions may improve hand perfusion while maintaining function of the access [1,5]. In the absence of correctable arterial lesions, surgical revascularization procedures such as distal revascularization with interval ligation or proximalization of arterial inflow may be indicated [5]. Overall, the management of DASS should prioritize preserving the access when possible while restoring adequate hand perfusion [5,9]. However, access ligation or embolization may be necessary in cases of severe, limb-threatening ischemia when access salvage is not feasible [5,7].

## Conclusions

DASS represents a hemodynamic imbalance between access flow and distal limb perfusion rather than a uniform clinical entity. This case illustrates that steal physiology may become clinically apparent or worsen following repeated endovascular interventions, even in the absence of proximal inflow disease. However, one limitation of this report is the absence of DBI measurement and digital pressures to formally grade severity. Furthermore, although our angiographic findings of distal hypoperfusion with flow reversal into the fistula strongly supported our diagnosis, it is important to note that this single case report cannot establish causality between endovascular intervention and symptom progression. Careful post-intervention reassessment of access hemodynamics should be performed, particularly in patients who develop new or worsening symptoms during dialysis.
